# Total magnesium intake and risk of frailty in older women

**DOI:** 10.1002/jcsm.13450

**Published:** 2024-06-06

**Authors:** Ellen A. Struijk, Teresa T. Fung, Heike A. Bischoff‐Ferrari, Walter C. Willett, Esther Lopez‐Garcia

**Affiliations:** ^1^ Department of Preventive Medicine and Public Health, School of Medicine Universidad Autónoma de Madrid‐IdiPaz Madrid Spain; ^2^ CIBERESP (CIBER of Epidemiology and Public Health) Madrid Spain; ^3^ Department of Nutrition Simmons University Boston MA USA; ^4^ Department of Nutrition Harvard T.H. Chan School of Public Health Boston MA USA; ^5^ Department of Geriatrics and Aging Research University Hospital Zürich and University of Zürich Zürich Switzerland; ^6^ Centre on Aging and Mobility University Hospital Zürich and Waid City Hospital Zürich Switzerland; ^7^ Channing Division of Network Medicine, Department of Medicine Brigham and Women's Hospital and Harvard Medical School Boston MA USA; ^8^ IMDEA/Food Institute, CEI UAM + CSIC Madrid Spain

**Keywords:** ageing, frailty, magnesium, nutrition, physical function

## Abstract

**Background:**

An adequate magnesium intake might lower the risk of frailty through its role in muscle function.

**Methods:**

We analysed data from 81 524 women aged ≥60 years participating in the Nurses' Health Study. Total magnesium intake was obtained from repeated food frequency questionnaires administered between 1984 and 2010 and self‐reported information on supplementation. Frailty was defined as having at least three of the following five FRAIL scale criteria: fatigue, low strength, reduced aerobic capacity, having ≥5 chronic illnesses and weight loss ≥ 5%. The occurrence of frailty was assessed every 4 years from 1992 to 2018. Cox proportional hazards models adjusted for lifestyle factors, medication use and dietary factors were used to assess the association between magnesium intake and frailty.

**Results:**

During a median follow‐up of 16 years, we identified 15 477 incident cases of frailty. Women with a higher intake of total energy‐adjusted magnesium had a decreased risk of frailty after adjustment for lifestyle factors, medication use and dietary factors. The relative risk (95% confidence interval) for Quintile 5 (Q5) versus Quintile 1 (Q1) was 0.88 (0.82, 0.94) (*P*‐trend < 0.001). When only energy‐adjusted magnesium from the diet was considered, the inverse association was stronger (Q5 vs. Q1: 0.68 [0.56, 0.82]; *P*‐trend < 0.001). Those reaching the recommended daily allowance (RDA) of magnesium through diet had a 14% (9%, 19%) lower risk of frailty compared with those not meeting the RDA.

**Conclusions:**

Increased intake of foods rich in magnesium was associated with a decreased risk of frailty.

## Introduction

Frailty is a geriatric syndrome characterized by a reduction in functioning across multiple physiological systems, which increases an individual's vulnerability to stressors. Once a person is frail, other geriatric syndromes are more likely to emerge, such as falls, disability and cognitive impairment, resulting in an increased likelihood of lowered quality of life, hospitalization and premature mortality.^1^, ^2^ Effective strategies that target the prevention and management of frailty in an ageing population would reduce the burden at both the individual and health system levels.

Magnesium is a mineral important in numerous intracellular physiological functions. As a cofactor, magnesium plays a crucial role in all enzymes utilizing or synthesizing muscle adenosine triphosphate (ATP) and indirectly in the muscle contraction and relaxation processes.^3^ Symptoms of magnesium deficiency include muscle cramps, fatigue and weakness.^3^ Older adults are at increased risk of magnesium deficiency due to insufficient dietary intake, decreased absorption and increased excretion, possibly aggravated by medication use, which may lower magnesium stores.^4^

An adequate magnesium intake might lower the risk of frailty through its beneficial role in muscle function. Evidence from studies on magnesium and physical performance is scarce and conflicting. In a randomized controlled trial among healthy older women, oral supplementation with 300 mg of magnesium for 12 weeks improved physical performance.^5^ Also, a prospective study showed that men, but not women, with a higher magnesium intake were less likely to become frail after 8 years of follow‐up,^6^ while another study found that an increase in magnesium intake over 5 years was associated with an improvement in physical performance, only in women.^7^ These population studies did not consider the impact of magnesium supplementation. Therefore, the aim of this study was to investigate the association between dietary and supplemental magnesium intake and the risk of frailty in a large population of older women.

## Methods

### Study design and participants

The Nurses' Health Study (NHS) cohort was established in 1976 with the enrolment of 121 700 female nurses aged 30–55 years at inception.^8^ Participants completed biennial mailed questionnaires to update information on medical history and lifestyle. The follow‐up rate was approximately 90% at each follow‐up cycle. The study protocol was approved by the Institutional Review Boards of the Brigham and Women's Hospital and the Harvard T.H. Chan School of Public Health.

For this analysis, we included women aged ≥60 years at the analytical baseline in 1990 with complete information on the exposure and outcome variables. Women younger than 60 years contributed person‐time to this analysis when they turned 60 during subsequent questionnaire cycles. Women with an implausible high (>3500 kcal/day) or low (<500 kcal/day) energy intake were excluded, as were women identified as frail at the analytical baseline, leaving a total population of 81 524 women for the main analysis (*Figure* *S1*). The association between magnesium consumption and frailty occurrence was examined up to 2018.

### Dietary assessment

Dietary intake was assessed using a validated food frequency questionnaire (FFQ) in 1984, 1986, 1990, 1994, 1998, 2000, 2006 and 2010, as described in detail elsewhere.^9^ In each questionnaire, participants were asked how often, on average, during the previous year they consumed the foods specified. A standard portion size and nine possible responses for the frequency of consumption, ranging from ‘never, or less than once per month’ to ‘six or more times per day’, were given for each food item. The intake of specific nutrients (including magnesium) was calculated by multiplying the frequency of consumption of each food recorded by its nutrient content using the US Department of Agriculture database, complemented with information from the manufacturers and summed across all foods. Women were additionally asked whether they took magnesium supplements. Although there was no information on the exact magnesium content of these supplements, the intake was estimated based on the most frequently used magnesium supplement on the market in the year of the questionnaires. The use of specific brands and types of multivitamins was ascertained, as was the number of supplements taken weekly. Total magnesium intake consisted of a combination of dietary and supplemental magnesium.

Nutrient intakes correlated with total energy intake (including total and dietary magnesium) were adjusted for total energy intake using the residual method.^10^ Macronutrients are presented as a percentage of total daily energy. The reproducibility and validity of the FFQs have been reported in detail elsewhere.^11^, ^12^ Briefly, the Spearman correlation coefficient between magnesium intake estimated from the FFQ and the average of two 7‐day dietary records was 0.73.^11^ To reduce within‐person variation and best represent the long‐term effects of magnesium intake, we used the cumulative average of total and dietary magnesium intake from all available dietary questionnaires from 1984 through frailty onset or the end of follow‐up.^13^ For example, the average magnesium intake of 1984, 1986 and 1990 was used to predict frailty occurrence from 1992 to 1996, and the average of 1984, 1986, 1990 and 1994 intake was used to predict risk from 1996 to 2000, and so on. Because of the strong increase in supplement intake over the years (in 1984, 38% of the women took multivitamins or magnesium supplements vs. 69% in 2010), we used the most recent supplemental magnesium intake before frailty onset or the end of follow‐up instead of the cumulative average intake over the years.

### Frailty assessment

We used the FRAIL scale,^14^ which includes five self‐reported frailty criteria: fatigue, low strength, reduced aerobic capacity, having several chronic illnesses and significant unintentional weight loss. In 1992, 1996, 2000, 2004, 2008, 2012 and 2016, participants completed the Medical Outcomes Study Short‐Form (SF‐36), a 36‐item questionnaire with eight health dimensions, including physical and mental components.^15^ From the SF‐36, we assessed the first three frailty criteria with the following questions: (a) for fatigue: ‘Did you have a lot of energy?’, with response ‘a little of the time’ or ‘none of the time’ (in 1992, 1996 and 2000), or with the question ‘I could not get going’ (in 2004), with response ‘moderate amount’ or ‘all of the time’, or with the question ‘I feel full of energy’ (in 2008, 2012 and 2016), with response ‘no’; (b) for low strength: ‘In a normal day, is your health a limitation to walk up 1 flight of stairs?’, with response ‘yes, limited a lot’; and (c) for reduced aerobic capacity: ‘In a normal day, is your health a limitation to walk several blocks or several miles?’, with response ‘yes, a lot’. In addition, the illness criterion was assessed by the question, ‘In the last 2 years, have you had any of these physician‐diagnosed illnesses?’. We considered that this criterion was met when participants reported ≥5 of the following diseases: cancer, hypertension, type 2 diabetes, angina, myocardial infarction, stroke, congestive heart failure, asthma, chronic obstructive lung disease, arthritis, Parkinson's disease, kidney disease and depression. Lastly, because the weight of the participants was available only biannually, the weight loss criterion was defined as a 5% decrease in the weight reported in a 2‐year period before the assessment of frailty. At the end of each 4‐year follow‐up cycle, incident frailty was defined as having ≥3 criteria on the scale. Missing responses in three or more components were assumed to be missing on frailty status and excluded. For those participants with one or two missing responses, we were able to define a frailty case with the existence of the other three available criteria. The FRAIL scale has been correlated (*r* = 0.62, *P* < 0.001) with the physical frailty phenotype,^16^ the most widely used scale for frailty assessment, which includes both self‐reported and performance‐based measures.

### Ascertainment of mortality

Deaths were reported by the next of kin, or the postal system, or ascertained through the National Death Index. Follow‐up for mortality was more than 98% complete.^17^ We obtained copies of death certificates and medical records to determine causes of death (classified according to the categories of the International Classification of Diseases, Ninth Revision). Death records were reviewed and coded by physicians.

### Socio‐economic variables, medical history, anthropometric data and lifestyle factors

In the analytic baseline questionnaire (1990), we collected information on age, indicators of socio‐economic status (education level and census track income), weight, smoking status and medication use. This information has been updated on each of the subsequent biennial questionnaires. To calculate body mass index (BMI), we used information on height reported in 1976, when the cohort was initiated, and updated self‐reported weight; BMI was calculated as weight in kilograms divided by the square of height in metres. Discretionary physical activity was reported as the average time spent per week during the preceding year in specific activities (e.g., walking outdoors, jogging and bicycling). The time spent in each activity was multiplied by its typical energy expenditure, expressed in metabolic equivalent tasks and then summed over all activities. Detailed information on the validity and reproducibility of self‐reported weight and physical activity has been published elsewhere.^18^, ^19^

### Statistical analysis

Participants were classified into quintiles of total magnesium, dietary magnesium and supplemental magnesium intake. We used Cox proportional hazards models to calculate relative risks (RRs), estimated by hazard ratios, and their 95% confidence interval (CI) for the studied associations, adjusting for potential confounders updated at each 4‐year cycle. Person‐years were calculated from the analytical baseline in 1990 until the occurrence of frailty, death or the end of the study period (1 June 2018), whichever came first. We stratified the analysis jointly by age in months at the start of follow‐up and the calendar year of each questionnaire cycle.

Multivariable models were adjusted for census tract income (<$45 000, $45 000–$59 999, $60 000–$74 999, $75 000–$99 999 or ≥$100 000/year), education (registered nursing degrees, bachelor's degree, masters or doctorate degree), BMI (<25.0, 25.0–29.9 and ≥30.0 kg/m^2^), smoking status (never, past and current: 1–14, 15–24 and ≥25 cigarettes/day), alcohol intake (0, 1.0–4.9, 5.0–14.9 and ≥15.0 g/day), energy intake (quintiles of kcal/day) and medication use (yes/no) including postmenopausal hormone therapy, aspirin, diuretics, beta‐blockers, calcium channel blockers, angiotensin‐converting enzyme inhibitors, other antihypertensive medication, lipid‐lowering medication, insulin and oral hypoglycaemic medication. Medication use was included in the model to address the fact that people with risk factors for chronic diseases are possibly at greater risk of developing frailty, although some over‐adjustment might exist. Similarly, because the inclusion of BMI might also represent some over‐adjustment, as weight loss is part of the frailty outcome, BMI was not updated, and only BMI measured at baseline was included in the analysis. Results were further adjusted for dietary factors by including multivitamin use, cereal fibre, calcium, protein, saturated fat, and sugar‐sweetened beverages in the model (quintiles). Models with supplemental magnesium were additionally adjusted for magnesium from the diet. As physical activity is closely related to the outcome, adjustment for this variable was only done in secondary analyses. Tests for linear trends were conducted by modelling the median intakes of each quintile as a continuous variable.

In addition, several sensitivity analyses were performed. We calculated the risk of frailty among those who reached the recommended daily allowance (RDA) of magnesium versus those who did not meet this recommendation.^20^ Only 23 women exceeded the tolerable upper intake level (UL) of magnesium intake by consuming more than 350 mg of magnesium from supplements a day; therefore, the impact of extremely high levels of magnesium in relation to frailty was not assessed. The association between magnesium intake and each criterion of the FRAIL scale was assessed. Additionally, analyses were stratified by diet quality expressed as adherence to the Alternate Healthy Eating Index (AHEI) score (below vs. above the median) and physical activity (below vs. above the median).^21^ We replicated the main analyses among those with 0 (robust) or 1–2 (prefrail) of the frailty criteria at baseline. Additionally, analyses were repeated, excluding women with diabetes, cardiovascular disease or cancer at baseline or those who developed these diseases during the follow‐up to assess the independence of this association from the main chronic diseases. All statistical tests were two‐sided with a *P* value < 0.05 and performed using SAS software, Version 9.4 for UNIX (SAS Institute Inc., Cary, NC, USA).

## Results

The cumulative average daily intake of total magnesium over the entire follow‐up period was 326 mg; on average, 298 mg/day came from the diet and 28 mg/day from supplements. Women in the highest quintile of total magnesium intake had a lower BMI, were more physically active, were less often current smokers and had a higher education level compared with those in the lowest quintile (*Table* *1*). There was no clear trend of medication use over the quintiles of magnesium intake except for postmenopausal hormone therapy being higher among those with a high magnesium intake. Furthermore, women with a high magnesium intake also had a higher intake of calcium, total protein and cereal fibre, with a lower intake of saturated fat, sugar‐sweetened beverages and alcohol.

**Table 1:** Baseline characteristics according to quintiles of energy‐adjusted total magnesium intake among women aged ≥60 years in the Nurses' Health Study

	Total magnesium				
	Quintile 1	Quintile 2	Quintile 3	Quintile 4	Quintile 5
Participants, *n*	18 448	17 252	16 430	15 152	14 242
Mean age, years	62.4 (2.2)	62.5 (2.2)	62.6 (2.3)	62.7 (2.3)	62.9 (2.4)
BMI, kg/m^2^	26.0 (5.1)	25.8 (4.8)	25.6 (4.6)	25.5 (4.6)	25.2 (4.5)
Discretionary physical activity, METs‐h/week	14.3 (20.0)	17.0 (20.5)	19.2 (22.2)	21.2 (23.8)	24.7 (28.2)
Current smoker, %	16	13	11	11	9
Education graduate school, %	2	2	3	3	4
Census tract income above 100 000/year, %	22	23	23	23	23
Medication use^a^					
Aspirin, %	42	46	48	48	48
Postmenopausal hormone therapy, %	32	36	38	40	43
Diuretics, %	10	10	10	10	10
Beta‐blockers, %	14	13	13	13	12
Calcium channel blockers, %	9	10	10	10	9
ACE inhibitors, %	10	10	10	10	9
Other blood pressure medication, %	9	9	9	9	8
Lipid‐lowering medication, %	16	17	16	16	15
Insulin, %	1	2	2	2	2
Oral hypoglycaemic drugs, %	3	3	3	3	3
Supplement use					
Magnesium supplement, %	1	2	4	7	15
Multivitamin supplement, %	32	44	52	59	71
Dietary intake					
Total magnesium, mg/day	238 (23.9)	282 (12.1)	311 (11.7)	344 (13.9)	411 (48.9)
Dietary magnesium, mg/day	233 (23.7)	272 (16.6)	296 (20.2)	320 (25.8)	360 (50.3)
Supplemental magnesium, mg/day	4.49 (10.7)	9.32 (16.1)	15.3 (21.6)	23.9 (28.0)	49.2 (46.2)
Calcium, mg/day	807 (313)	956 (321)	1062 (345)	1171 (369)	1368 (429)
Protein, % of energy	16.9 (2.45)	18.0 (2.32)	18.6 (2.41)	19.1 (2.51)	19.8 (2.84)
Saturated fat, % of energy	12.1 (2.14)	11.5 (1.91)	11.0 (1.91)	10.4 (1.87)	9.61 (1.97)
Cereal fibre, g/day	3.83 (1.30)	4.48 (1.54)	4.97 (1.80)	5.57 (2.14)	6.62 (3.24)
Sugar‐sweetened beverages, servings/day	0.47 (0.63)	0.26 (0.37)	0.19 (0.30)	0.15 (0.26)	0.11 (0.20)
Energy intake, kcal/day	1726 (467)	1764 (454)	1771 (448)	1773 (441)	1722 (448)
Alcohol intake, g/day	6.47 (10.8)	6.37 (9.72)	6.12 (8.97)	5.63 (8.3)	4.85 (7.36)
Number of frailty criteria, %					
0	68	70	71	73	73
1	25	23	23	22	21
2	8	7	6	6	5

*Note*: Values are means (SD) unless otherwise indicated. All data, except age, were directly standardized to the age distribution of the entire cohort.

Abbreviations: ACE, angiotensin‐converting enzyme; BMI, body mass index; METs, metabolic equivalent tasks.

^a^ One or more times per week.

During a median follow‐up of 16 years, we identified a total of 15 477 incident cases of frailty. After adjusting for age, lifestyle factors, medication use and dietary covariates, we observed a significant inverse association between total magnesium intake and risk of frailty (Quintile 5 [Q5] vs. Quintile 1 [Q1], RR [95% CI]: 0.88 [0.82, 0.94]; *P*‐trend < 0.001) (*Table* *2*). When only dietary magnesium was considered, the inverse association was stronger (Q5 vs. Q1: 0.68 [0.56, 0.82]; *P*‐trend < 0.001). Women in the highest quintile of supplemental magnesium did not have a significant lower risk of frailty after adjustment for lifestyle factors, medication use and dietary factors (Q5 vs. Q1: 0.96 [0.90, 1.02]; *P*‐trend = 0.96). Also, each 200‐mg increase in dietary magnesium intake was significantly associated with a lower risk of frailty (0.53 [0.40, 0.70]), while a 200‐mg increase in supplemental magnesium did not increase the risk of frailty (1.00 [0.96 1.05]) (data not shown in tables). Additional adjustments for physical activity did not change the results (*Table* *S1*).

**Table 2:** Relative risks (95% confidence interval) of frailty according to quintiles of magnesium intake among women aged ≥60 years in the Nurses' Health Study

	Magnesium categories					*P* value
	Quintile 1	Quintile 2	Quintile 3	Quintile 4	Quintile 5	
Total magnesium						
Magnesium range, mg/day	≤256	257–288	289–318	319–356	≥357	
Person‐years	262 610	264 009	264 240	264 068	264 832	
Frailty cases, *n*	3315	3103	3019	3026	3014	
Age adjusted	1.00	0.89 (0.84, 0.93)	0.82 (0.78, 0.86)	0.77 (0.73, 0.81)	0.73 (0.70, 0.77)	<0.001
Multivariable model^a^	1.00	0.91 (0.86, 0.95)	0.86 (0.82, 0.91)	0.82 (0.78, 0.86)	0.81 (0.77, 0.85)	<0.001
Multivariable model^b^	1.00	0.94 (0.89, 0.99)	0.91 (0.86, 0.96)	0.87 (0.82, 0.93)	0.88 (0.82, 0.94)	<0.001
Dietary magnesium						
Magnesium range, mg/day	≤245	246–274	275–300	301–332	≥333	
Person‐years	64 922	65 418	65 526	65 244	65 627	
Frailty cases, *n*	534	453	467	448	397	
Age adjusted	1.00	0.82 (0.72, 0.93)	0.76 (0.67, 0.87)	0.72 (0.63, 0.82)	0.58 (0.51, 0.66)	<0.001
Multivariable model^a^	1.00	0.78 (0.68, 0.89)	0.77 (0.67, 0.87)	0.73 (0.64 0.83)	0.61 (0.53, 0.70)	<0.001
Multivariable model^b^	1.00	0.80 (0.70, 0.92)	0.80 (0.68, 0.92)	0.77 (0.65, 0.90)	0.68 (0.56, 0.82)	<0.001
Supplemental magnesium						
Magnesium range, mg	0	0.01–1.30	1.31–50	51–75	≥76	
Person‐years	148 596	273 558	180 641	220 763	175 714	
Frailty cases, *n*	3403	1807	1244	3004	2090	
Age adjusted	1.00	0.93 (0.86, 1.00)	0.92 (0.86, 0.98)	0.91 (0.87, 0.96)	0.92 (0.87, 0.97)	0.07
Multivariable model^a^	1.00	0.93 (0.86, 1.00)	0.95 (0.89, 1.01)	0.94 (0.89, 0.99)	0.96 (0.90, 1.01)	0.64
Multivariable model^b^	1.00	0.93 (0.86, 1.01)	0.95 (0.89, 1.02)	0.94 (0.88, 0.99)	0.96 (0.90, 1.02)	0.96

^a^ Cox regression model adjusted for age (months), calendar time (4‐year intervals), census tract income (<$45 000, $45 000–$59 999, $60 000–$74 999, $75 000–$99 999 or ≥$100 000/year), education (registered nursing degrees, bachelor's degree, masters or doctorate degree), baseline body mass index (<25.0, 25.0–29.9 and ≥30.0 kg/m^2^), smoking status (never, past and current: 1–14, 15–24 and ≥25 cigarettes/day), alcohol intake (0, 1.0–4.9, 5.0–14.9 or ≥15.0 g/day), energy intake (quintiles of kcal/day) and medication use (aspirin, postmenopausal hormone therapy, diuretics, beta‐blockers, calcium channel blockers, angiotensin‐converting enzyme inhibitors, other blood pressure medication, lipid‐lowering medication, insulin and oral hypoglycaemic medication).

^b^ Adjustment as in the previous model and additionally adjusted for multivitamin use, cereal fibre, calcium, protein, saturated fatty acids and sugar‐sweetened beverages (quintiles). Models for supplemental magnesium were additionally adjusted for magnesium from the diet.

Only 52% of the women met the RDA for total magnesium intake of ≥320 mg/day (*Table* *3*). Compared with those not meeting the RDA, women with a dietary magnesium intake above the RDA had a 14% (9%, 19%) lower risk of frailty; women who reached the RDA with additional supplemental magnesium intake had a 4% lower risk of developing frailty compared with those not reaching the RDA; however, this association did not reach significance.

**Table 3:** Relative risks (95% confidence interval) of frailty according to meeting or not meeting the recommended daily allowance of cumulative magnesium intake, with and without supplements

	Not meeting the RDA^a^	Meeting the RDA with magnesium from the diet^b^	Meeting the RDA only in combination with supplements^c^
Participants, *n*	39 539	35 164	6821
Person‐years	574 057	586 829	158 872
Frailty cases, *n*	6834	6304	2339
Age adjusted	1.00	0.85 (0.82, 0.88)	0.95 (0.91, 1.00)
Multivariable model^d^	1.00	0.78 (0.75, 0.82)	0.94 (0.90, 0.99)
Multivariable model^e^	1.00	0.86 (0.81, 0.91)	0.96 (0.91, 1.01)

Abbreviation: RDA, recommended daily allowance.

^a^ <320 mg/day of total magnesium.

^b^ ≥320 mg/day of total magnesium and ≥320 mg/day of dietary magnesium.

^c^ ≥320 mg/day of total magnesium and <320 mg/day of dietary magnesium.

^d^ Cox regression model adjusted for age (months), calendar time (4‐year intervals), census tract income (<$45 000, $45 000–$59 999, $60 000–$74 999, $75 000–$99 999 or ≥$100 000/year), education (registered nursing degrees, bachelor's degree, masters or doctorate degree), baseline body mass index (<25.0, 25.0–29.9 and ≥30.0 kg/m^2^), smoking status (never, past and current: 1–14, 15–24 and ≥25 cigarettes/day), alcohol intake (0, 1.0–4.9, 5.0–14.9 or ≥15.0 g/day), energy intake (quintiles of kcal/day) and medication use (aspirin, postmenopausal hormone therapy, diuretics, beta‐blockers, calcium channel blockers, angiotensin‐converting enzyme inhibitors, other blood pressure medication, lipid‐lowering medication, insulin and oral hypoglycaemic medication).

^e^ Adjustment as in the previous model and additionally adjusted for multivitamin use, cereal fibre, calcium, protein, saturated fatty acids and sugar‐sweetened beverages (quintiles).

The intake of total magnesium was significantly associated with two of the five frailty criteria: fatigue and the weight loss criterion (*Table* *S2*). Dietary magnesium was associated with having five or more diseases and the weight loss criterion, while supplemental magnesium intake was not associated with any of the frailty criteria. Stratification by diet quality showed that the association between total magnesium intake and frailty held only among those with a low‐quality diet (*Table* *S3*), although there was no significant interaction between diet quality and magnesium in association with frailty. Also, those with a high physical activity level seemed to benefit more from a higher total and dietary magnesium intake, but no significant interaction was detected (*Table* *S4*). The association between total magnesium and frailty was attenuated among prefrail women at baseline (*Table* *S5*). Lastly, the association found between magnesium intake and frailty did not change when analyses were performed among those without cancer, diabetes or heart disease (*Table* *S6*).

## Discussion

In this large cohort study of older women, we found that total magnesium and particularly dietary magnesium intake were inversely associated with the development of frailty; magnesium supplementation was not associated with a lower risk of frailty. Women meeting the RDA of magnesium intake from their diet had a 14% lower risk of developing this syndrome. These results were independent of other lifestyle factors, medication use and several dietary factors.

So far, only two other prospective studies have explored the association between magnesium intake and physical performance. In a study among 4421 participants from North America with or at high risk of osteoarthritis, higher dietary magnesium was associated with a lower risk of frailty in men but not in women after 8 years of follow‐up.^6^ In addition, among 863 older adults from a study in Spain, an increment in magnesium intake was associated with improved physical function, as approached with the lower extremity functional scale, among women but not among men, in a 5‐year period.^7^ Additionally, two large cross‐sectional studies with participants from the EPIC‐Norfolk and UK Biobank cohorts found an association between dietary magnesium and muscle mass in both men and women.^22^, ^23^

In line with the previous studies, we found an association between dietary magnesium and frailty, but not for supplemental magnesium intake. A possible explanation could be that women with high consumption of supplements may already have deteriorated functional status. However, when excluding women who were prefrail or with cancer, diabetes or heart disease at baseline, the association between supplemental magnesium and frailty remained non‐significant. In contrast, in a randomized controlled trial among 139 healthy older women attending a mild fitness programme, results showed that those who were supplemented with 300 mg/day of magnesium had significantly better physical performance after 12 weeks of intervention than the control group.^5^ These findings were more evident in participants with a dietary magnesium intake lower than the RDA. In our study, we found that among women who did not reach the RDA through the diet alone, those who reached the magnesium RDA with additional supplemental intake did not have a significantly lower risk of frailty compared with those who did not reach the RDA.

Recent data from the National Health and Nutrition Examination Survey (NHANES) showed that 63% of the women aged 71 years and older did not reach an adequate amount of magnesium intake.^24^ Similarly, in our sample, 52% of the women did not reach the RDA. However, it should be noted that the RDA is set to meet the requirements of 98% of the population, so for some women, 320 mg/day of magnesium is more than they need. For older adults for whom healthy eating can be a challenge due to dental problems, chewing difficulties, high costs of healthy foods or loss of appetite,^25^ a multivitamin supplement including magnesium could be of benefit to reaching an adequate nutrient intake. For most older adults, an optimal palatable diet pattern that provides enough minerals as well as an optimal macronutrient composition, favouring nutrient absorption, is likely to help prevent the development of geriatric syndromes.^26^

Magnesium is available in the diet in a variety of foods. The most important sources of dietary magnesium in the NHS population were whole grains, fortified cereals, milk, coffee, nuts and beans. Several other foods that are high in magnesium, including green leafy vegetables, have previously been associated with a lower risk of frailty.^6^, ^27^ Therefore, we cannot exclude that other beneficial components of these foods are partly responsible for the inverse association found.

The exact mechanisms through which magnesium may impact physical function and muscle performance are not entirely clear. A higher magnesium intake has previously been shown to be significantly related to higher bone mineral density in older White women and men.^28^ Interactions between bone and muscle through multiple physiological pathways, including hormonal and inflammatory pathways, are thought to contribute to the onset of frailty.^29^ Additionally, an independent relationship was found between serum magnesium levels with muscle performance and several muscle parameters in older men and women.^30^ Magnesium has a key role in the production of muscle energy through magnesium ions utilizing or synthesizing muscle ATP. Magnesium deficiency can cause fatigue and weakness, conditions often followed by poor muscle function.^3^ Our results showed an association between total magnesium intake and the fatigue component of frailty. Fatigue is typically one of the first criteria that manifests while developing physical frailty.^31^ Additionally, low magnesium intake has been associated with several adverse health effects, including an increased production of free radicals and increased levels of inflammation markers,^3^, ^32^, ^33^, ^34^ factors that have been related to chronic diseases such as cancer, heart disease and diabetes, as well as frailty.^35^, ^36^ In our analysis, dietary magnesium was associated with the multimorbidity component of frailty; nonetheless, sensitivity analyses among a subgroup of women without these diseases showed that even though frailty and several chronic diseases share common pathways, this pathway did not fully explain the inverse association found between magnesium and frailty.

A major strength of this study is the large sample size and the use of repeated dietary assessments and updated information on covariates over 28 years of follow‐up. However, several limitations need to be acknowledged. First, we cannot exclude that other nutrients present in food products rich in magnesium are partly responsible for the beneficial association found, although we adjusted for the most relevant potentially beneficial nutrients, including calcium, protein and cereal fibre. Second, as dietary information was self‐reported and the amount of supplemental magnesium was estimated, measurement error and misclassification could occur. However, the FFQ used has been extensively validated against diet records and biomarkers, showing a good correlation for total magnesium intake.^11^ Third, only one definition of frailty was used, and thus, our results should be confirmed in studies using other definitions, such as the frailty phenotype.^1^ The FRAIL scale may have lower accuracy compared with a frailty definition that does not solely include self‐reported measures. However, performance‐based measures are not feasible in a large cohort such as the NHS, and although we were able to adjust for many potential confounders, residual and unmeasured confounding cannot be completely ruled out. Finally, the possibility of reverse causation cannot be totally discarded because frailty might develop gradually and therefore affect dietary habits.

In conclusion, our findings suggest that women with a high intake of foods rich in magnesium have a lower risk of frailty. A diet that provides enough magnesium to comply with the RDA seems like an optimal strategy to prevent frailty syndrome. Some of the benefit observed may partially be due to other favourable nutrients in foods high in magnesium.

## Conflict of interest statement

All authors declare that they have no conflict of interest.

## Supporting information


**Figure S1.** Participants flow chart.
**Table S1.** Relative risks (95% confidence interval) of frailty according to quintiles of magnesium intake among women aged ≥60y in the Nurses' Health Study, additionally adjusted for physical activity.
**Table S2.** Relative risks (95% confidence interval) of frailty criteria according to quintiles of magnesium intake among women aged ≥60y in the Nurses' Health Study.
**Table S3.** Relative risks (95% confidence interval) of frailty according to quintiles of magnesium intake among women aged ≥60y in the Nurses' Health Study, stratified by diet quality.
**Table S4.** Relative risks (95% confidence interval) of frailty according to quintiles of magnesium intake among women aged ≥60y in the Nurses' Health Study, stratified by physical activity (METs‐h/wk).
**Table S5.** Relative risks (95% confidence interval) of frailty according to quintiles of magnesium intake among women robust or prefrail at baseline.
**Table S6.** Relative risks (95% confidence interval) of frailty according to quintiles of magnesium intake among women without diabetes, heart disease and cancer.
