# Supplementary material for: Total magnesium intake and risk of frailty in older women
Source: J Cachexia Sarcopenia Muscle. 2024 Jun 6;15(4):1275–82. doi: 10.1002/jcsm.13450 (PMC11294045; doi:10.1002/jcsm.13450)
Supplement: Supplementary file 1 — Figure S1. Participants flow chart. Table S1. Relative risks (95% confidence interval) of frailty according to quintiles of magnesium intake among women aged ≥60y in the Nurses' Health Study, additionally adjusted for physical activity. Table S2. Relative risks (95% confidence interval) of frailty criteria according to quintiles of magnesium intake among women aged ≥60y in the Nurses' Health Study. Table S3. Relative risks (95% confidence interval) of frailty according to quintiles of magnesium intake among women aged ≥60y in the Nurses' Health Study, stratified by diet quality. Table S4. Relative risks (95% confidence interval) of frailty according to quintiles of magnesium intake among women aged ≥60y in the Nurses' Health Study, stratified by physical activity (METs‐h/wk). Table S5. Relative risks (95% confidence interval) of frailty according to quintiles of magnesium intake among women robust or prefrail at baseline. Table S6. Relative risks (95% confidence interval) of frailty according to quintiles of magnesium intake among women without diabetes, heart disease and cancer. [file JCSM-15-1275-s001.docx]

**Total magnesium intake and risk of frailty in older women**

Ellen A. Struijk, Teresa T. Fung, Heike A. Bischoff-Ferrari, Walter C. Willett, Esther Lopez-Garcia.

Table of contents

**Figure S1. Participants flow chart.**

**Table S1**. Relative risks (95% confidence interval) of frailty according to quintiles of magnesium intake among women aged ≥60y in the Nurses’ Health Study, additionally adjusted for physical activity.

**Table S2**. Relative risks (95% confidence interval) of frailty criteria according to quintiles of magnesium intake among women aged ≥60y in the Nurses’ Health Study.

**Table S3** Relative risks (95% confidence interval) of frailty according to quintiles of magnesium intake among women aged ≥60y in the Nurses’ Health Study, stratified by diet quality.

**Table S4**. Relative risks (95% confidence interval) of frailty according to quintiles of magnesium intake among women aged ≥60y in the Nurses’ Health Study, stratified by physical activity (METs-h/wk).

**Table S5** Relative risks (95% confidence interval) of frailty according to quintiles of magnesium intake among women robust or prefrail at baseline.

**Table S6**. Relative risks (95% confidence interval) of frailty according to quintiles of magnesium intake among women without diabetes, heart disease and cancer.

Nurses´ Health Study

n=121,700

**Figure S1. Participant flow chart**

Final sample

n= 81,524 women were followed-up until 2018

Exclusions

- Women younger than 60 years at baseline (1990) are not included until they turn 60 at a subsequent follow-up cycle.
- Women without a food frequency questionnaire or with an unreasonably high (>3500 kcal/d) or low (<500 kcal/d) caloric intake.
- Women that died before baseline.
- Women identified as frail at baseline.

| **Table S1**. Relative risks (95% confidence interval) of frailty according to quintiles of magnesium intake among women aged ≥60y in the Nurses’ Health Study, additionally adjusted for physical activity. | | | | | | |
| --- | --- | --- | --- | --- | --- | --- |
|  | Magnesium categories | | | | |  |
|  | Quintile 1 | Quintile 2 | Quintile 3 | Quintile 4 | Quintile 5 | P value |
| Total magnesium | 1.00 | 0.96 (0.91, 1.01) | 0.94 (0.89, 0.99) | 0.91 (0.85, 0.97) | 0.92 (0.86, 0.99) | 0.02 |
| Dietary magnesium | 1.00 | 0.83 (0.72, 0.96) | 0.84 (0.72, 0.98) | 0.82 (0.69, 0.96) | 0.75 (0.61, 0.91) | 0.01 |
| Supplemental magnesium | 1.00 | 0.93 (0.86, 1.00) | 0.95 (0.89, 1.02) | 0.94 (0.88, 0.99) | 0.96 (0.90, 1.02) | 0.91 |
| ^1^Cox regression model adjusted for: age (months), calendar time (4-y intervals), census tract income (<$45,000, $45,000–$59,999, $60,000–$74,999, $75,000–$99,999, or ≥$100,000/y), education (registered nursing degrees, bachelor’s degree, masters or doctorate degree), baseline body mass index (<25.0, 25.0-29.9, ≥30.0 kg/m^2^), smoking status (never, past, and current 1-14, 15-24, and ≥25 cigarettes/day), alcohol intake (0, 1.0-4.9, 5.0-14.9, or ≥15.0 g/d), energy intake (quintiles of kcal/d), medication use (aspirin, postmenopausal hormone therapy, diuretics, β-blockers, calcium channel blockers, ACE inhibitors, other blood pressure medication, lipid lowering medication, insulin, and oral hypoglycemic medication), multivitamin use, cereal fiber, calcium, protein, saturated fatty acids, sugar sweetened beverages, and physical activity (quintiles of METs- h/wk). Models for supplemental magnesium were additionally adjusted for magnesium from the diet. | | | | | | |

| **Table S2**. Relative risks (95% confidence interval) of frailty criteria according to quintiles of magnesium intake among women aged ≥60y in the Nurses’ Health Study. | | | | | | |
| --- | --- | --- | --- | --- | --- | --- |
|  | Magnesium categories | | | | |  |
|  | Quintile 1 | Quintile 2 | Quintile 3 | Quintile 4 | Quintile 5 | P value |
| **Fatigue** |  |  |  |  |  |  |
| Total magnesium | 1.00 | 0.98 (0.90, 1.07) | 0.92 (0.84, 1.02) | 0.88 (0.79, 0.97) | 0.87 (0.77, 0.97) | 0.005 |
| Dietary magnesium | 1.00 | 0.94 (0.75, 1.16) | 0.86 (0.68, 1.09) | 0.81 (0.62, 1.05) | 0.77 (0.57, 1.04) | 0.06 |
| Supplemental magnesium | 1.00 | 0.94 (0.83, 1.06) | 0.89 (0.78, 1.00) | 0.91 (0.82, 1.01) | 0.93 (0.84, 1.04) | 0.36 |
| **Low strength** |  |  |  |  |  |  |
| Total magnesium | 1.00 | 0.99 (0.91, 1.07) | 1.00 (0.92, 1.09) | 0.94 (0.85, 1.03) | 0.98 (0.88, 1.08) | 0.53 |
| Dietary magnesium | 1.00 | 0.83 (0.68, 1.03) | 0.89 (0.71, 1.10) | 0.90 (0.71,1.14) | 0.90 (0.68, 1.18) | 0.67 |
| Supplemental magnesium | 1.00 | 0.95 (0.85, 1.05) | 1.00 (0.90, 1.12) | 0.98 (0.89, 1.07) | 0.96 (0.87, 1.06) | 0.64 |
| **Low aerobic capacity** |  |  |  |  |  |  |
| Total magnesium | 1.00 | 0.96 (0.87, 1.05) | 0.92 (0.84, 1.02) | 0.86 (0.78, 0.96) | 0.92 (0.82, 1.04) | 0.13 |
| Dietary magnesium | 1.00 | 0.98 (0.77, 1.24) | 0.94 (0.72, 1.21) | 0.84 (0.63, 1.11) | 0.82 (0.59, 1.13) | 0.16 |
| Supplemental magnesium | 1.00 | 0.98 (0.87, 1.11) | 0.92 (0.81, 1.05) | 0.91 (0.82, 1.01) | 0.95 (0.85, 1.06) | 0.76 |
| **≥5 diseases** |  |  |  |  |  |  |
| Total magnesium | 1.00 | 0.88 (0.77, 1.00) | 0.88 (0.76, 1.01) | 0.90 (0.77, 1.05) | 0.83 (0.70, 0.99) | 0.08 |
| Dietary magnesium | 1.00 | 0.98 (0.67, 1.41) | 0.69 (0.46, 1.04) | 0.57 (0.36, 0.89) | 0.64 (0.38, 1.09) | 0.03 |
| Supplemental magnesium | 1.00 | 0.92 (0.76, 1.11) | 1.03 (0.86, 1.22) | 0.87 (0.75, 1.01) | 0.92 (0.79, 1.08) | 0.83 |
| **Weight loss** |  |  |  |  |  |  |
| Total magnesium | 1.00 | 0.99 (0.90, 1.08) | 0.91 (0.82, 1.01) | 0.86 (0.77, 0.96) | 0.85 (0.75, 0.96) | 0.003 |
| Dietary magnesium | 1.00 | 0.77 (0.61, 0.98) | 0.77 (0.60, 0.99) | 0.78 (0.59, 1.02) | 0.65 (0.47, 0.90) | 0.02 |
| Supplemental magnesium | 1.00 | 0.97 (0.86, 1.10) | 0.98 (0.87, 1.12) | 0.94 (0.84, 1.05) | 1.00 (0.89, 1.12) | 0.83 |
| ^1^Cox regression model adjusted for: age (months), calendar time (4-y intervals), census tract income (<$45,000, $45,000–$59,999, $60,000–$74,999, $75,000–$99,999, or ≥$100,000/y), education (registered nursing degrees, bachelor’s degree, masters or doctorate degree), baseline body mass index (<25.0, 25.0-29.9, ≥30.0 kg/m^2^), smoking status (never, past, and current 1-14, 15-24, and ≥25 cigarettes/day), alcohol intake (0, 1.0-4.9, 5.0-14.9, or ≥15.0 g/d), energy intake (quintiles of kcal/d), medication use (aspirin, postmenopausal hormone therapy, diuretics, β-blockers, calcium channel blockers, ACE inhibitors, other blood pressure medication, lipid lowering medication, insulin, and oral hypoglycemic medication), multivitamin use, cereal fiber, calcium, protein, saturated fatty acids, and sugar sweetened beverages (quintiles). Models for supplemental magnesium were additionally adjusted for magnesium from the diet. | | | | | | |

| **Table S3** Relative risks (95% confidence interval) of frailty according to quintiles of magnesium intake among women aged ≥60y in the Nurses’ Health Study, stratified by diet quality. | | | | | | | |
| --- | --- | --- | --- | --- | --- | --- | --- |
|  | Magnesium categories | | | | | |  |
|  | Quintile 1 | Quintile 2 | Quintile 3 | Quintile 4 | Quintile 5 | | P value |
| **Total magnesium** |  |  |  |  |  | |  |
| Low AHEI score (<median) | 1.00 | 0.92 (0.87, 0.98) | 0.92 (0.86, 0.98) | 0.87 (0.81, 0.94) | 0.97 (0.89, 1.06) | 0.02 | |
| High AHEI score (≥median) | 1.00 | 0.99 (0.87, 1.12) | 0.94 (0.83, 1.06) | 0.94 (0.84, 1.06) | 0.92 (0.82, 1.04) | | 0.10 |
| **Dietary magnesium** |  |  |  |  |  | |  |
| Low AHEI score (<median) | 1.00 | 0.83 (0.71, 0.96) | 0.82 (0.70, 0.96) | 0.94 (0.78, 1.13) | 0.74 (0.57, 0.94) | | 0.02 |
| High AHEI score (≥median) | 1.00 | 0.93 (0.64, 1.34) | 0.94 (0.66, 1.32) | 0.86 (0.61, 1.20) | 0.78 (0.56, 1.09) | | 0.03 |
| **Supplemental magnesium** | |  |  |  |  | |  |
| Low AHEI score (<median) | 1.00 | 0.89 (0.81, 0.99) | 0.99 (0.91, 1.08) | 0.91 (0.85, 0.98) | 0.96 (0.89, 1.04) | | 0.72 |
| High AHEI score (≥median) | 1.00 | 0.99 (0.88, 1.12) | 0.92 (0.83, 1.02) | 1.00 (0.92, 1.09) | 1.03 (0.95, 1.13) | | 0.27 |
| ^1^Cox regression model adjusted for: age (months), calendar time (4-y intervals), census tract income (<$45,000, $45,000–$59,999, $60,000–$74,999, $75,000–$99,999, or ≥$100,000/y), education (registered nursing degrees, bachelor’s degree, masters or doctorate degree), baseline body mass index (<25.0, 25.0-29.9, ≥30.0 kg/m^2^), smoking status (never, past, and current 1-14, 15-24, and ≥25 cigarettes/day), alcohol intake (0, 1.0-4.9, 5.0-14.9, or ≥15.0 g/d), energy intake (quintiles of kcal/d) and medication use (aspirin, postmenopausal hormone therapy, diuretics, β-blockers, calcium channel blockers, ACE inhibitors, other blood pressure medication, lipid lowering medication, insulin, and oral hypoglycemic medication), and multivitamin use. Models for supplemental magnesium were additionally adjusted for magnesium from the diet. | | | | | | | |

| **Table S4**. Relative risks (95% confidence interval) of frailty according to quintiles of magnesium intake among women aged ≥60y in the Nurses’ Health Study, stratified by physical activity (METs-h/wk). | | | | | | |
| --- | --- | --- | --- | --- | --- | --- |
|  | Magnesium categories | | | | |  |
|  | Quintile 1 | Quintile 2 | Quintile 3 | Quintile 4 | Quintile 5 | P value |
| **Total magnesium** |  |  |  |  |  |  |
| Low physical activity (<median) | 1.00 | 0.93 (0.87, 0.99) | 0.92 (0.86, 0.99) | 0.92 (0.85, 0.99) | 0.95 (0.87, 1.04) | 0.31 |
| High physical activity (≥median) | 1.00 | 1.00 (0.91, 1.09) | 0.93 (0.85, 1.03) | 0.87 (0.78, 0.96) | 0.88 (0.78, 0.98) | 0.01 |
| **Dietary magnesium** |  |  |  |  |  |  |
| Low physical activity (<median) | 1.00 | 0.81 (0.68, 0.96) | 0.76 (0.63, 0.92) | 0.86 (0.69, 1.06) | 0.75 (0.58, 0.97) | 0.05 |
| High physical activity (≥median) | 1.00 | 0.79 (0.61, 1.04) | 0.87 (0.66, 1.15) | 0.72 (0.53, 0.97) | 0.64 (0.46, 0.90) | 0.01 |
| **Supplemental magnesium** | |  |  |  |  |  |
| Low physical activity (<median) | 1.00 | 0.90 (0.82, 1.00) | 0.95 (0.86, 1.04) | 0.91 (0.84, 0.99) | 0.95 (0.87, 1.03) | 0.58 |
| High physical activity (≥median) | 1.00 | 0.96 (0.85, 1.09) | 0.94 (0.83, 1.05) | 0.96 (0.87, 1.05) | 0.96 (0.87, 1.06) | 0.52 |
| ^1^Cox regression model adjusted for: age (months), calendar time (4-y intervals), census tract income (<$45,000, $45,000–$59,999, $60,000–$74,999, $75,000–$99,999, or ≥$100,000/y), education (registered nursing degrees, bachelor’s degree, masters or doctorate degree), baseline body mass index (<25.0, 25.0-29.9, ≥30.0 kg/m^2^), smoking status (never, past, and current 1-14, 15-24, and ≥25 cigarettes/day), alcohol intake (0, 1.0-4.9, 5.0-14.9, or ≥15.0 g/d), energy intake (quintiles of kcal/d) and medication use (aspirin, postmenopausal hormone therapy, diuretics, β-blockers, calcium channel blockers, ACE inhibitors, other blood pressure medication, lipid lowering medication, insulin, and oral hypoglycemic medication)^,^ multivitamin use, cereal fiber, calcium, protein, saturated fatty acids, and sugar sweetened beverages (quintiles). | | | | | | |

| **Table S5** Relative risks (95% confidence interval) of frailty according to quintiles of magnesium intake among women robust or prefrail at baseline. | | | | | | |
| --- | --- | --- | --- | --- | --- | --- |
|  | Magnesium categories | | | | |  |
|  | Quintile 1 | Quintile 2 | Quintile 3 | Quintile 4 | Quintile 5 | P value |
| **Robust** |  |  |  |  |  |  |
| Total magnesium | 1.00 | 0.94 (0.87, 1.02) | 0.90 (0.82, 0.97) | 0.85 (0.77, 0.93) | 0.85 (0.77, 0.95) | 0.001 |
| Dietary magnesium | 1.00 | 0.94 (0.74, 1.18) | 0.78 (0.61, 1.01) | 0.63 (0.48, 0.84) | 0.70 (0.51, 0.96) | 0.01 |
| Supplemental magnesium | 1.00 | 1.00 (0.90, 1.12) | 1.00 (0.90, 1.11) | 0.95 (0.87, 1.03) | 0.93 (0.85, 1.02) | 0.22 |
| **Prefrail** |  |  |  |  |  |  |
| Total magnesium | 1.00 | 0.97 (0.89, 1.07) | 0.93 (0.85, 1.03) | 0.92 (0.82, 1.02) | 0.94 (0.84, 1.06) | 0.32 |
| Dietary magnesium | 1.00 | 0.69 (0.54, 0.88) | 0.60 (0.46, 0.79) | 0.61 (0.45, 0.81) | 0.46 (0.32, 0.65) | <0.001 |
| Supplemental magnesium | 1.00 | 0.92 (0.81, 1.03) | 0.96 (0.84, 1.08) | 0.96 (0.86, 1.07) | 0.99 (0.88, 1.10) | 0.71 |
| ^1^Cox regression model adjusted for: age (months), calendar time (4-y intervals), census tract income (<$45,000, $45,000–$59,999, $60,000–$74,999, $75,000–$99,999, or ≥$100,000/y), education (registered nursing degrees, bachelor’s degree, masters or doctorate degree), baseline body mass index (<25.0, 25.0-29.9, ≥30.0 kg/m^2^), smoking status (never, past, and current 1-14, 15-24, and ≥25 cigarettes/day), alcohol intake (0, 1.0-4.9, 5.0-14.9, or ≥15.0 g/d), energy intake (quintiles of kcal/d), medication use (aspirin, postmenopausal hormone therapy, diuretics, β-blockers, calcium channel blockers, ACE inhibitors, other blood pressure medication, lipid lowering medication, insulin, and oral hypoglycemic medication), multivitamin use, cereal fiber, calcium, protein, saturated fatty acids, and sugar sweetened beverages (quintiles). Models with supplemental magnesium were additionally adjusted for magnesium from the diet. | | | | | | |

| **Table S6**. Relative risks (95% confidence interval) of frailty according to quintiles of magnesium intake among women without diabetes, heart disease and cancer. | | | | | | | |
| --- | --- | --- | --- | --- | --- | --- | --- |
|  | Magnesium categories | | | | | |  |
|  | Quintile 1 | | Quintile 2 | Quintile 3 | Quintile 4 | Quintile 5 | P value |
| Total magnesium | | 1.00 | 0.93 (0.86, 1.00) | 0.90 (0.83, 0.97) | 0.82 (0.75, 0.90) | 0.80 (0.73, 0.89) | <0.001 |
| Dietary magnesium | | 1.00 | 0.80 (0.66, 0.97) | 0.76 (0.61, 0.93) | 0.67 (0.53, 0.84) | 0.59 (0.45, 0.78) | <0.001 |
| Supplemental magnesium | | 1.00 | 0.92 (0.83, 1.01) | 0.94 (0.85, 1.04) | 0.93 (0.86, 1.01) | 0.92 (0.84, 1.00) | 0.47 |
| ^1^Cox regression model adjusted for: age (months), calendar time (4-y intervals), census tract income (<$45,000, $45,000–$59,999, $60,000–$74,999, $75,000–$99,999, or ≥$100,000/y), education (registered nursing degrees, bachelor’s degree, masters or doctorate degree), baseline body mass index (<25.0, 25.0-29.9, ≥30.0 kg/m^2^), smoking status (never, past, and current 1-14, 15-24, and ≥25 cigarettes/day), alcohol intake (0, 1.0-4.9, 5.0-14.9, or ≥15.0 g/d), energy intake (quintiles of kcal/d), medication use (aspirin, postmenopausal hormone therapy, diuretics, β-blockers, calcium channel blockers, ACE inhibitors, other blood pressure medication, lipid lowering medication, insulin, and oral hypoglycemic medication), multivitamin use, cereal fiber, calcium, protein, saturated fatty acids, and sugar sweetened beverages (quintiles). Models with supplemental magnesium were additionally adjusted for magnesium from the diet. | | | | | | | |
